# Development and Validation of the Intimate Partner Violence Workplace Disruptions Assessment (IPV-WDA)

**DOI:** 10.3390/ijerph22071147

**Published:** 2025-07-19

**Authors:** Kathryn Showalter, Laneshia Conner, Rebecca Bosetti, William Burrows, Rujeko Machinga-Asaolu

**Affiliations:** 1 College of Social Work, University of Kentucky, Lexington, KY 40508, USA; laneshia.conner@uky.edu (L.C.); rebecca.bosetti@uky.edu (R.B.); 2 Department of Biostatistics, University of Kentucky, Lexington, KY 40508, USA; burton.burrows@uky.edu; 3 School of Social Work, Boise State University, Boise, ID 83725, USA; rujekomachinga@boisestate.edu

**Keywords:** intimate partner violence, abuser-initiated workplace disruptions, workplace responses, technology harassment, measurement development

## Abstract

A vast majority of survivors of intimate partner violence (IPV) experience economic abuse, including but not limited to, employment sabotage. The purpose of this study is to further understand IPV by testing a technology-inclusive abuser-initiated workplace disruption measurement in an exploratory factor analysis (EFA) so that future researchers can better examine and address economic abuse. Using a sample of survivors (N = 312) employed in the nursing profession in the United States, who may be uniquely impacted by technology, we used complete data to examine experiences of abuser-initiated workplace disruptions, including those that utilized cellphones (e.g., excessive texting, harassment of coworkers, preventing educational advancement). The results revealed a two-factor structure: one containing a variety of direct and indirect workplace disruptions relevant to the nursing profession (73% of variance) and a second containing only cell-phone related harassment (9% of variance). Implications for healthcare employers seeking to protect employees from IPV, as well as policymakers, are included.

## 1. Introduction

Intimate partner violence (IPV) is a national public health crisis, impacting at least 40% of women in the United States during their lifetimes [1]. While there are many forms of IPV, technology-facilitated abuse in relationships (TAR) is a relatively new pattern of abuse that is widely engaged in [2]. As a form of coercive control through the misuse of technology, TAR uses commonly deployed sexual, emotional, psychological, and economic abuse behaviors, yet it has distinct behaviors that are unique to digital platforms (e.g., online stalking, checking mobile phones without permission, impersonation, sharing material with consent, etc.) [3]. With the rapid expansion of technology and its devices, there has been a rapid growth in its weaponization within relationships. Considering the detriment of these actions, TAR is difficult to detect and prove for survivors. Given its unique features (e.g., no geographical borders, ability to transcend time, omnipresence), it is a distinct form of control and should be considered in the broader context of IPV. Hence, attempts to address this issue have risen over the past decade to regulate and prevent its occurrence.

In the past decade, scholars have developed scales to measure TAR across a range of behaviors, digital devices, and applications [2,4,5]. Of note are recommendations to create measures that span across behaviors as well as individuals. By exploring TAR in other contexts, such as in the workplace, there is potential to understand more about the likelihood of victimization in roles that occupy a key amount of time. This is especially true for employees who use their personal cellphones to communicate with coworkers. Certain professions, like healthcare or specifically nursing, have replaced pagers with cellphones and texting to communicate between hospital floors, wings, and patient rooms [6,7]. To understand the issue of TAR more fully in the workplace, an understanding of economic abuse is required.

### 1.1. Disruptions in the Workplace

IPV survivors can gain economic stability through their employment, making the workplace a target of destruction for abusive partners [8]. Employment has been shown to meet basic needs, improve psychosocial functioning, and importantly, establish financial independence. A major barrier to leaving an abusive situation is financial dependence from the abuser, especially when survivors have become dependent on the abuser [9]. As cited by Gonzalez [10], the findings from the Economic Security for Survivors project indicate that 73% of IPV survivors stayed with their abusive partners because of financial reasons. Economic abuse manifests in one or more of the following three behaviors: (1) control of financial resources, (2) manipulating current financial situations and (3) sabotaging current and prospective employment [11]. An estimated 93% of all IPV survivors experience economic abuse or abusive behaviors that control, restrict, prevent, or sabotage their financial resources or credit [12,13,14,15]. Economic abuse behaviors, also known as employment sabotage, occur in the form of abuser-initiated workplace disruptions, such as threats through text messaging or impairing authorized functions [2,14]. Economic abuse is frequently invisible; however, when asked about types of IPV experienced, this hidden abuse is one of the highest reported among IPV survivors.

As with other forms of hidden abuse, economic abuse comes with a complexity of adverse outcomes for survivors and their families. Specifically, economic abuse among intimate partners is associated with shortened life spans, a decline in physical and emotional health, poor quality of life, negative parenting practices, and harmful youth outcomes [16,17,18]. Economic abuse is also associated with a decrease in economic self-sufficiency among survivors [19,20]. In a study of diverse low-income survivors, economically controlling behaviors and economic sabotage by male partners were unique predictors of decreased economic self-sufficiency [19]. Likewise, experiencing any form of economic control significantly predicted a decrease in economic self-sufficiency [20]. Of particular interest to the authors is when perpetrators specifically sabotage survivors’ employment.

### 1.2. Employment Sabotage

Employment sabotage includes preventing a survivor from obtaining or maintaining employment, harassing a survivor at their place of work, and forbidding or interfering with a survivor’s employment or education for promotion [21,22]. In a study of IPV survivors who were participating in a financial literacy program (N = 120), 78% of the sample experienced employment sabotage [20]. Likewise, 88% of IPV survivors in a subsequent study had experienced employment sabotage [11]. The Economic Security for Survivors project [10] found that more than half of the survivors lost their jobs due to the abusive conduct of their partners. In the same sample, 70% of participants were prohibited by their partner from working, and 78% were prohibited from enrolling in school, missing out on earning a credential that could secure higher-wage jobs [10]. As indicated by a literature review (N = 35) [17], abuser-initiated workplace disruptions also have severe health impacts on survivors. IPV survivors miss hours, days, weeks, and many moments of concentration at work when they are experiencing abuse [23]. Abusive partners directly interfere in their victims’ employment (e.g., deleting electronic calendar appointments or physically preventing them from sleeping) as well as indirectly (e.g., belittling confidence or threatening to leave children alone during the workday) [8,24]. Additionally, employment sabotage is positively associated with psychosocial health issues [25], including poorer mental health [26], like depressive symptoms [19]. The effects of IPV on employment have been found to have a lagged effect, resulting in unemployment as many as six years after the abuse has occurred [12,27,28]. Researching the sabotage of employment for IPV survivors is crucial to informing the development of effective interventions, policies, and support systems for survivors.

Given the unique spatial dynamics that make economic abuse unique to other forms of abuse, it is critical to incorporate economic abuse broadly in IPV measurements, especially since the recent growth in the literature available [29]. The first and most frequently used IPV measurement tool is the Conflict Tactics Scale (CTS; [30]), and was established in the 1970s. The CTS was used significantly more often than any other measurement and is largely used in North America to examine violence against women [31]. In this same scoping review of over 1000 studies, researchers found that the Abuse Assessment Screen (AAS) in clinical settings and the World Health Organization Violence Against Women Survey (WHO) in population/community settings were both used in about 10% of studies sampled [31]. As commonly used as the measures were, the CTS, CTS-2, and AAS do not have economic violence-specific items, and the WHO survey asks only about being prevented from working and spending money.

### 1.3. Measures of Economic Abuse

Historically, economic abuse was measured as a form of psychological abuse, capturing economically abusive behaviors yet not defining them as being a distinct dimension of victimization [32,33]. Measurement of economic abuse in IPV relationships was generally not specific to workplace disruptions. Even though they are financially focused, the Domestic Violence Related Financial Issues Scale [34,35] and the Scale of Economic Abuse [21] do not specifically measure economic abuse as it relates to employment. This has changed, as research has focused on specific dimensions of economic abuse and the workplace, investigating access to money, credit rating, debt, and other controlling behaviors that involve money and finances [36]. For example, items such as “I’ve been prevented from working” or “I’ve been prevented from spending money” can be found on the Abuse Behavior Inventory [32], the Index of Spousal Abuse [37], the Psychological Maltreatment of Women Inventory [33], and the Checklist of Controlling Behaviors [38].

Financial harm through the use of technology has also grown, to help account for the ways that technology can be used to exploit, disadvantage, and control survivor’s access to and use of financial resources. Recent investigations [4,5] have produced and tested instruments and scales that analyze cyber dating violence, and better capture the diversity of perpetrator tactics to understand and improve measures of IPV. In addition to these measures, the Work/School Abuse Scale (W/SAS; [39]) and The Work-Related Control, Abuse, and Sabotage Checklist (WORCASC; Brush, [40]) ask about preventing employment altogether and abusive tactics that are used before, after, and during work hours. Further, the WORSASC asks a series of innovative items about work-related control that indicate high internal consistency (α = 0.92) but does not account for technological advancements that give abusive partners additional ways to wreak havoc on IPV survivors’ lives (e.g., identity-related crimes, account compromise, destroying physical records or property, etc., [41]). Thus, the Intimate Partner Violence- Workplace Disruptions Assessment (IPV-WDA) tested in this analysis is the first IPV-related economic abuse measurement tool focusing solely on employment sabotage with technology items. Of particular interest to the authors were to examine the growing threat of technology-facilitated gender-based violence, and to look more closely at this phenomenon among the female-dominated profession, nursing.

### 1.4. Nursing and Technology-Facilitated IPV

In the U.S., women make up most nursing professionals [42] and individuals who experience intimate partner violence (IPV; [43]). While it is estimated that 40% of the general population of U.S. women experience IPV in their lifetimes, 65% of all nurses have suffered IPV or physical, sexual, economic, mental, and emotional abusive behaviors used by a partner to exert power and control [44,45]. In addition, nurses experience severe challenges in the workplace. After the 2020 pandemic, nurses’ stress and burnout rates are extremely high [46,47]. Recently, due to COVID-19, nurses have shown more signs of depression and anxiety than before [48]. Given the historical association of violence with depression, anxiety, and PTSD [49], mental health problems are likely compounded for nurses experiencing IPV. In the case of nurses, the burden of unpredictable schedules and extended hours of work [50,51] leads to stress and a strain on familial relationships [52] and possibly IPV [27].

Perpetrators are using technology to control, monitor, threaten, impersonate, and harass those they abuse who work in medical fields [53]. While mass replacement of pagers with smartphones in medical settings has made improvements in workflow efficiency and communication [6,7], they pose a threat to nurses’ concentration when abusive partners use them for harassment [8]. However, there is minimal information about technology-facilitated IPV and subsequent measurements to understand these tactics to which nurses might be especially vulnerable.

Due to the extremely delicate job tasks of nurses that require remembering a vast amount of patient data and staying focused during a crisis, being distracted by abusive partners at work may be of greater risk not only to the IPV survivor but also to the patients they see [8]. Nurses who are IPV survivors might be more likely to have their employment directly sabotaged because of the security of the job and the potential for upward mobility in large hospitals [54].

## 2. Current Study

Considering the unique situation to female nurses and the workplace context of technology-facilitated IPV, this paper describes the validation of a new instrument, the Intimate Partner Violence- Workplace Disruptions Assessment (IPV-WDA). The IPV-WDA focuses on employment sabotage, and specifically includes items relevant to the occurrence of technology-facilitated abuse among individuals in female-dominated professions. The current study utilizes an exploratory factor analysis (EFA), recommended for scale development and evaluation [55] to test a technology-inclusive measurement of economic and employment abuse among nurses. The analysis is theory-generating and helps determine factors important to our pre-screened and tested workplace disruption items [55,56]. While previous research has used factor analysis for economic abuse scales like the SEA-12 [11], the current study aims to fill these gaps in the literature on the measurement of technology-facilitated abuse in a female-dominated profession such as nursing. The goals of the present study were the following:

1. Determine the factor structure of the IPV-WDA scale through EFA and demonstrate reliability of the IPV-WDA scale.

2. Describe the relationships between items and unique factors based on data from 312 nurses identified by the EFA.

## 3. Materials and Methods

### 3.1. Sample and Design

Our study was guided by previous scale development in the field of technology-facilitated IPV see [4,5,39,40]. This cross-sectional study of nurses’ self-reported experiences of IPV was conducted between April and October of 2021 in one southern State in the U.S. Participants were recruited from statewide membership organizations specific to nursing, such as unions and professional development chapters. An advertisement for the study was sent by listservs and/or to the home addresses of organization members. The study was conducted anonymously and entirely online to enhance participants’ safety and mitigate health concerns. In addition, all participants were provided with a list of local, state, and national resources for survivors of IPV. The fully online survey took 35–45 min and contained items on intimate partner violence, workplace disruptions, demographics, workplace support for IPV, and employment outcomes (e.g., hours worked, days worked, job status). Participants were offered an e-gift card for their time, and a link external to their survey responses was provided to submit a secure email address. The study received full consideration and approval of the appropriate Institutional Review Board.

The survey consisted of two portions that participants completed based on eligibility. Participants were eligible for the first portion of the study if they were employed as a nurse during the COVID-19 pandemic (fall of 2020 and beyond) and eligible for the second portion if they had at any point in their nursing career, experienced IPV. Participants were considered to have experienced IPV if they marked “yes” to the question, “Have you ever in your lifetime experienced intimate partner violence (IPV or physical, mental, emotional, economic, or sexually abusive and controlling behaviors by a romantic partner)?” Further, participants were eligible for the second portion of the study if they agreed with the statement, “Have you experienced IPV while you were employed as a Nurse in the state of ‘state name’?” For the current study, we selected a subsample of participants that had complete data on the IPV questionnaire and demographic information (N = 312).

### 3.2. Measurement

The Intimate Partner Violence and Workplace Disruptions Assessment (IPV-WDA; [23]) is a comprehensive measurement of abusive tactics that partners use to sabotage survivors’ employment directly (See Table 1). The measurement development process has followed the best practices recommended by experts (see [57]). The IPV-WDA was created using survivors’ accounts of abuse and a review of existing measures [58]. Following this qualitative analysis, the measure was pilot-tested with survivors receiving care at a midwestern shelter [57]. Pilot testing showed excellent psychometric properties regarding construct validity and overall reliability (r = 0.968) [23,58,59]. Thus far, in the development of the measurement, all items have been shown to reveal unique information about the construct of IPV-WDA and thus have been retained [23].

Experiences of abuser-initiated workplace disruptions were measured with the current sample using 38 items. Items were drawn from the original piloted measure of the IPV-WDA [23] and four additional items that emerged in consultation with experts. The IPV-WDA is a comprehensive measurement designed to include technology-related abuse tactics that could be used to harass IPV survivors in the workplace. Example items include “My partner called my cell phone many times a day while I was at work.” Participants endorsed the frequency of each tactic from never (0) to always (4).

### 3.3. Data Analysis

Descriptive statistics were generated to describe the sample. Data was assessed for missingness and found to be missing at random. Approximately 6% of cases contained missing data, and as such, a complete case analysis was performed. Studies suggest that a complete case analysis is unbiased when data is missing at random around 5% of cases are missing [60,61]. EFA is a widely used analytic method to take observed variables, identify their correlations, and connect them to conceptual latent variables [62]. When no known prior theory is identified for the data structure, EFA is preferred to a confirmatory factor analysis (CFA). EFA is often the first step in scale development and is subsequently followed up by CFA in a separate sample.

Analysis: Process Description. The data was assessed for univariate and multivariate normality using the Shapiro–Wilk normally test and z-kurtosis, respectively (see Appendix A). Data was non-normally distributed. Therefore, principal axis factoring (PAF) was used as the extraction method [63,64,65]. To test data suitability for analysis, Kaiser-Meyer-Olkin’s measure of sampling adequacy and Bartlet’s test of sphericity were generated for the correlation matrix. An overall measure of sampling adequacy of 0.98 and a *p*-value < 0.05 for the test of sphericity indicate that the data is acceptable to analyze.

To assess the number of factors in our data we used Kaiser’s eigenvalue > 1 rule, Cattell’s Scree Test, and Parallel Analysis. The results of Kaiser’s eigenvalue > 1 rule and Cattell’s Scree Test indicate that two factors would be appropriate for our data. However, the results of parallel analysis suggest that three factors would be appropriate. We tested both two and three factors and found that no items loaded onto a third factor regardless of rotational method, indicating that two factors are appropriate for our data [66]. An oblique rotation method was chosen for this analysis to generate correlations between factors, which is impossible with an orthogonal rotation method [67]. Recent simulation studies suggest that when performing an EFA, researchers should perform factor rotations from multiple starting points and compare at least two different rotation methods [68].

We performed an exploratory factor analysis on participants (n = 312) using a PAF extraction with factor rotations from 2000 random starting points and compared the results between an Oblimin rotation method and GeominQ rotation method [69]. Results were similar between rotational methods, but local fit was better with the Oblimin rotation method; all results presented are from the Oblimin rotation method.

Item relationships were assessed using a factor loading cutoff of 0.7 and communality value of >0.5 [69]. Questions were removed stepwise based on factor loading cutoffs and communality values. After each question removal, a new EFA was performed with the remaining questions, and new factor loading cutoffs and communality values were generated. Cronbach alphas were generated for each factor to assess internal consistency. All statistical analyses were completed in R 4.2.2. [70].

## 4. Results

### 4.1. Sample Characteristics

Participants were, on average, 33.1 (SD = 9.5) years old and had 1.1 (SD = 1.0) children. A majority of participants identified as female (86.7%), white (82.3%), and non-Hispanic (68.9%). Participants had a range of educational attainments, including an associate’s degree (30.4%) and a bachelor’s degree in nursing (16.7%). Complete sample characteristics are presented in Table 2.

### 4.2. EFA Results

Questions were eliminated one at a time in a stepwise manner in the following order: Q14, Q2, Q23, Q1, Q28, Q21, Q11, Q26, Q30, Q25, Q15, Q12, Q26, Q38, Q33, Q19, Q6, Q29, Q3, Q20, Q20, Q27. Questions were eliminated based on factor loadings of less than <0.7 and low communality [69]. A complete listing of factor loadings, communalities, and Cronbach Alphas is presented in Table 3. Of the original 38 questions, 22 were removed. Examples of removed items are “My partner monitored my work calendar or planner” and “My partner made me take frequent breaks or long lunch periods during work hours.” It is possible that work calendars are not as critical in nursing as in other professions that have to schedule meetings or organize collaborations. Similarly, nurses likely have set breaks, so it might not be more difficult to find a victim and take a break. The remaining 16 questions spread across two factors account for 73% of the variance in participants’ experiences of abuser-initiated workplace disruptions.

Factor one includes general abuser-initiated workplace disruptions (both direct and indirect created by abusers) that could happen before, after, or during work hours and accounted for 63.5% of the variance in experiences of abuser-initiated workplace disruptions. Factor two consisted of cellphone-based disruption tactics and accounted for 9.0% of the variance in experiences of abuser-initiated workplace disruptions.

## 5. Discussion

IPV is an issue that spans multiple levels of the social ecology, both in the perpetration of violence and the ramifications in the wake of enduring ongoing threats. The inception of the IPV-WDA came in response to our gap in understanding how abusive partners disrupt the employment of survivors using in-person and technology-facilitated tactics. The current study represents a critical step in establishing measurement construct validity by examining factor structure after the initial development of items through in-depth qualitative interviews with survivors and pilot testing of the scale assessed item relevance and validity [23]. Findings indicate that not only are workplace disruptions critical to study in order to understand the means to perpetrate and effects of intimate partner violence, but that workplace and economic abuse are not monolithic experiences. In particular, we observed subcategories of abuser-initiated direct and indirect tactics and a separate factor of cellphone-facilitated disruption tactics, suggesting the need to cast a wide net for capturing the nuances of employment and economic abuse IPV.

Our first factor of abuser-initiated workplace disruptions revealed important direct and indirect disruptions for nursing professionals. First, direct items that were important in predicting nurses’ experiences included sabotaging transportation to work and sabotaging education needs for promotion. Transportation might be essential for nurses who need to travel daily to centrally located places like hospitals or home health providers who need to travel to meet patients. Further, much of the “ladder climbing” in the nursing profession is based on degree obtainment. The difference in pay scale is about 30 K more for nurses with a bachelor’s degree vs. an associate’s degree [42]. Indirectly, nurses showed that apathy at work was one way that abusive-initiated workplace disruptions were exhibited during their workday. In this, nurses did not feel motivated to return from lunch periods or breaks when experiencing IPV, resulting in loss of work hours. It is also noteworthy that this factor included items like missing weeks of work or being fired/resigning that some early economic abuse researchers measured as outcomes (see [28,71]) and what others have referred to as “employment instability” [8,72]. It is essential to consider that abuser-initiated workplace disruptions may not just be about abusive behaviors but also the side effects of those behaviors (e.g., sporadic work history) [23].

Surprisingly, a second factor emerged, with just two items accounting for nearly 10% of the total variance in reported workplace disruptions. These items looked at cellphone facilitated disruptions, including repeated calls and text messages to the survivor at work. The emergence of a cellphone-specific factor is notable for several reasons. Firstly, this may have emerged as a predominant disruption as nurses may need to rely on their cell phones to perform job duties. Recent research has explored the mass replacement of pagers with smartphones in medical settings and the subsequent improvements in workflow efficiency, response time to emergency calls and patient needs, as well as quality of communication [6,7,73]. Nurses who must rely on their smartphones to improve their patient care and adhere to workplace standards for communication may be more susceptible to ongoing harassment and disruption through cell phones. Moreover, abusive partners who are aware of nurses’ reliance on this technology may intentionally engage in cellphone harassment with the knowledge that their partners will have no choice but to see the calls and messages in the regular performance of their job duties. Of note, other technology items were included in the measurement instrument, such as email and social media harassment, yet those items loaded onto the first factor. This suggests that there may be unique elements to cellphone harassment that are more salient to nurses or that cellphones are the current technology that provides greater or more disruptive access for perpetrators of IPV. Future studies must consider how emergent technologies shift the landscape across professions.

The literature indicates that direct in-person IPV predicts technology-facilitated IPV; this risk is exacerbated by the ubiquity of cellphone use in society [74]. Survivors have identified that they feel inadequately prepared to deal with abuse perpetrated using technology and highlight the complexities inherent to managing technology-facilitated abuse while also innately relying on those same devices to access children and social support [53]. The current study contributes to this research landscape in several important ways. While there is evidence indicating technology-facilitated IPV can impact social relationships and access to support, the present study constitutes some of the first evidence for how this form of abuse impacts workplace safety, job engagement, and economic independence and stability for survivors of IPV. A recent scoping review noted that there is insufficient availability and use of validated measurements instruments when studying technology-facilitated IPV [75], and the validation of the IPV-WDA represents a critical step forward in being able to rigorously explore the use of cellphones in the perpetration of IPV across spheres of influence in the life of the survivor. The same review revealed that the vast majority of studies on technology-facilitated IPV sample from adolescent and college student populations [75]. While we might expect that these younger groups to be disproportionately impacted by technology-facilitated IPV, the saturation of technology reliance across ages in social and professional settings necessitates that we reimagine technology-facilitated IPV as a universal rather than age-restricted concern, as demonstrated by the magnitude of impact on nurses in the current study.

The items eliminated in establishing the factor structure of the measure provide interesting commentary and important justification for the study IPV on unique sectors. The 19 items that were not included fell broadly into three categories. First were items that may have limited relevance due to lack of survivor awareness. For instance, items that asked if the abuser physically followed the survivor, waited outside of the work location of the survivor or monitored the survivor’s location or calendar indicate a subversive nature of abuse. While these instances may have occurred, they could have happened easily without the survivor’s awareness. Second, items may have been worded in a confusing or inapplicable way to participants. For instance, the question of whether the abuser disrupted the survivor’s morning routine may not be relevant or understood the same way by nurses who work irregular shifts or at night. Further, questions about whether nurses were denied promotions or met their long-term workplace goals could have irrelevant wording, as perhaps the other workplace disruptions caused by IPV precluded the survivors from ever being offered promotions or from feeling able to set long-term goals in their professional careers. Third, some items might not apply to the nursing profession. These items included forgetfulness, slow work pace, and not paying attention to detail. Any medical profession, such as nursing, inordinately relies on detail orientation and swiftness as patients’ lives depend on getting appropriate care in a timely manner; thus, we may be less likely to observe these impacts of workplace disruption for nurses specifically. Instead, when studying this group of professionals, questions regarding the impact on bedside manner, patient engagement, investment, or other emotional care aspects may be more relevant for nurses.

### 5.1. Limitations

The generalizability of the findings is limited for a few reasons. First, we must consider sampling and data concerns. The majority of the sample was female, White, Non-Hispanic, and comprised solely of those in the nursing profession. Therefore, findings may not represent the experiences of marginalized groups of IPV survivors like those who are transgender or nonbinary, Black and Brown identifying, multi-racial, as well as survivors with disabilities [76]. Specifically, research has indicated differences in workplace support between groups of Black and White IPV survivors. Participants were also drawn from a single geographical region, meaning the findings may not reflect regional cultural nuances regarding workplace disruptions. Specifically, many participants were employed in mid-sized cities in large hospitals that likely do not reflect the experiences of rural-dwelling or home healthcare nursing professionals. Moreover, while this study represents a significant step in validating the measurement instrument IPV-WDA, validation is an ongoing process that needs to be repeated with new samples in unique settings. The measurement development process is incomplete, and improvements are being made to the IPV-WDA in its current form. The next step with a nursing population would be to conduct a confirmatory factor analysis ahead of any inferential or predictive models. In contrast, unique samples would benefit from conducting a new EFA with the complete item list from the original measure to capture nuances specific to other professions.

### 5.2. Implications

Continued research in this area should explore how experiencing IPV threatens survivors’ work and economic stability. Too often, survivors’ work and employment experiences are not included in the research or, perhaps worse, are lumped together in categories of employment/unemployment with no variance in careers. Future measurement modifications must consider including profession-specific items and whether some original items may lack relevance for a given sample or population. It is important to realize that all items were developed based on the lived experiences of IPV survivors and were assessed and validated previously [57], so future research with distinct populations should include the entire battery of items and perform EFA to determine whether factor structure is stable or varies based on place of employment or chosen profession. It is possible the measurement could be used to assess the experiences of IPV survivors in similar helping professions, but future testing is needed to confirm. Another important next research step would be to examine the convergent validity of the IPV-WDA with the aforementioned measures of economic abuse to capture the nuances of these interdependent concerns and determine the predictive validity of the IPV-WDA on important health and safety outcomes for survivors of IPV. Finally, future studies of technology-facilitated IPV must employ living measures that are prepared to evolve with the technology landscape as platforms and mediums of access continue to advance and become the norm within society at large.

Healthcare organizations, particularly large hospital employers, should use the findings from the current study to create or improve upon existing policies for nurses. To start, zero-tolerance policies for IPV in the workplace [76] that imply mutual (victim and perpetrator) responsibility for bringing abuse to work are not feasible for nurses. For instance, based on current findings, nurses cannot simply keep their cellphones in a locker with other personal items at work as they need them to communicate with coworkers. Instead, a starting place for employers of nurses is to implement disruption-preventative policies to avoid frequently endorsed responses from participants (e.g., Missed weeks of work, Didn’t want to return to shift from breaks, quitting, or resigning). Such policies should consider requiring management to confidentially assess the well-being of all nurses during regular supervision (particularly by asking about home safety) and/or establishing organization-wide paid-time-off programs that allow employees to address stressful/family situations. Related research indicates that when employers and hospital executives take an interest in employees’ safety either by seeking out their feedback or creating employee wellness programs, nurses experience lower stress levels [76].

## 6. Conclusions

The current study contributes to the conceptualization and measurement of economic abuse from intimate partners, specifically as it applies to sabotaging employment. Previous factor analyses of economic abuse measurements have not been focused on abuser-initiated workplace disruptions, accounted for advancements in technology, or used data of specific careers. Findings that cellphone harassment at work is prevalent among nurses experiencing IPV should be focused on by healthcare administrators protecting employees. Future research should continue to focus on understanding the experiences of IPV survivors by profession for best responses and interventions in the workplace.

## Figures and Tables

**Table 1:** Thirty-eight-item IPV-WDA questionnaire.

	Never	Rarely	Sometimes	Often	Always
My partner physically followed me when I went to work.	1	2	3	4	5
My partner disrupted my morning routine making me late to work.	1	2	3	4	5
My partner sabotaged childcare arrangements when I needed to work.	1	2	3	4	5
**My partner sabotaged my work transportation.**	1	2	3	4	5
My partner made me take frequent breaks or long lunch periods during work.	1	2	3	4	5
My partner distracted me from working by waiting outside the place I work.	1	2	3	4	5
**My partner confronted/attacked me in the parking lot of my workplace.**	1	2	3	4	5
**My partner came to my job and harassed patients.**	1	2	3	4	5
**My partner came to my job and harassed my coworkers.**	1	2	3	4	5
**My partner called my cell phone many times a day while I was at work.**	1	2	3	4	5
My partner made unwanted phone calls to my office phone during work.	1	2	3	4	5
My partner sent unwanted messages to me on social media websites.	1	2	3	4	5
**My partner sent unwanted messages to my coworkers on social media.**	1	2	3	4	5
My partner monitored my location using websites or cell phone tracking while I was at work.	1	2	3	4	5
My partner monitored my work calendar or planner.	1	2	3	4	5
**My partner texted me many times a day while I was at work.**	1	2	3	4	5
**My partner emailed me many times a day while I was a work.**	1	2	3	4	5
**My partner sabotaged the education I need to be promoted.**	1	2	3	4	5
My partner sabotaged the professional relationships I need to be promoted.	1	2	3	4	5
My partner accused me of having romantic relationships with coworkers to keep me from spending time with them.	1	2	3	4	5
My partner wanted or demanded sex when I needed to do work/leave	1	2	3	4	5
**My partner physically prevented me from going to work/working.**	1	2	3	4	5
I had a noticeably slower pace when completing tasks at work.	1	2	3	4	5
**I didn’t care about doing well at my job.**	1	2	3	4	5
I did not feel confident at work.	1	2	3	4	5
I did not take promotions or opportunities for advancement at work.	1	2	3	4	5
I felt tired during the work day.	1	2	3	4	5
I forgot to do tasks at work or would have difficulty remembering what to do.	1	2	3	4	5
I did not pay attention to detail.	1	2	3	4	5
I had difficulty focusing at work.	1	2	3	4	5
**I lost hours of work coming late or leaving early.**	1	2	3	4	5
**I lost hours of work taking excessive breaks or long lunch periods.**	1	2	3	4	5
I missed days of work.	1	2	3	4	5
**I missed weeks of work (5 or more consecutive work days).**	1	2	3	4	5
**I was fired or laid off from my job.**	1	2	3	4	5
**I quit or felt forced to resign from my job.**	1	2	3	4	5
**I have a sporadic work history from the time I experienced abuse.**	1	2	3	4	5
I have not been able to meet long-term goals at work.	1	2	3	4	5

Note: Bolded items are retained by the EFA in the current study.

**Table 2:** Characteristics of IPV-WDA sample.

Characteristic ^1^	Total (N = 312)
Age, years	33.1 (9.5)
Household Size	3.6 (1.5)
Number of Children	1.1 (1.0)
Gender Identity	
Female	271 (86.7%)
Male	39 (12.5%)
Non-Gender Conforming	1 (0.003%)
Other	1 (0.003%)
Race ^2^	
Black or African American	49 (15.7%)
White	258 (82.3%)
Other	4 (1.3%)
Ethnicity ^3^	
Hispanic/Latinx	80 (25.6%)
Non-Hispanic/Non-Latinx	215 (68.9%)
Other	14 (4.5%)
Highest Level of Education ^4^	
Licensed Practical Nurse	24 (7.7%)
Associate Degree in Nursing	95 (30.4%)
Registered Nurse	110 (35.3%)
Bachelor’s Degree in Nursing	52 (16.7%)
Master’s Degree in Nursing	22 (7.5%)
Doctor of Nursing Practice	6 (1.9%)
PhD in Nursing Research	2 (0.6%)

^1^ Statistics are presented as mean (standard deviation) for continuous variables and no. (%) for categorical variables. ^2^ Missing: 1, ^3^ Missing: 3, ^4^ Missing: 1.

**Table 3:** Final generated factor structure.

Question	Factor 1	Factor 2	h^2^ Communality
Q4	0.814	0.077	0.741
Q7	0.820	0.070	0.744
Q8	0.953	−0.135	0.777
Q9	0.873	−0.034	0.729
Q10	0.086	0.781	0.696
Q13	0.909	−0.084	0.745
Q16	−0.004	0.828	0.681
Q17	0.725	0.148	0.672
Q18	0.743	0.089	0.636
Q22	0.811	0.071	0.730
Q24	0.770	0.055	0.646
Q31	0.784	0.102	0.718
Q32	0.864	0.052	0.801
Q34	0.898	−0.052	0.755
Q35	0.886	−0.032	0.753
Q36	0.850	0.025	0.748
Q37	0.892	−0.044	0.752
% of variance	63.5	9.0	72.5
Cronbach alpha	0.975	0.817	

Factor Correlation: Factor 1 ↔ Factor 2 r—0.582. Factor Loading coefficients greater than 0.7 were italicized and retained for that specific factor.

## Data Availability

The data presented in this study are available on request from the corresponding author. The data are not publicly available due to the risk of identification.

## Appendix A

**Table A1:** Correlation matrix assessment results.

Statistical Test		
Kaiser-Meyer-Olkin Measure of Sampling Adequacy		0.98
Bartletts Test of Sphericity	Approx. Chi-Square	12,546.64
	df	703
	Significance	0.000

## References

[1] Leemis R.W., Friar N., Khatiwada S., Chen M.S., Kresnow M.J., Smith S.G., Caslin S., Basile K.C. The National Intimate Partner and Sexual Violence Survey: 2016/2017 Report on Intimate Partner Violence. https://stacks.cdc.gov/view/cdc/124646.

[2] Fiolet R., Brown C., McKay D., Marsden S., Leins K., Harris B. (2023). Perpetrator perceptions on the emotions and motivations driving technology-facilitated abuse in relationships: A story completion study. J. Interpers. Violence.

[3] Fraser C., Olsen E., Lee K., Southworth C., Tucker S. (2010). The New Age of Stalking: Technological Implications for Stalking. Juv. Fam. Court. J..

[4] Brown C., Hegarty K. (2021). Development and validation of the TAR Scale: A measure of technology-facilitated abuse in relationships. Comput. Hum. Behav. Rep..

[5] Watkins L.E., Maldonado R.C., DiLillo D. (2016). The Cyber Aggression in Relationships Scale: A new multidimensional measure of technology-based intimate partner aggression. Assessment.

[6] Przybylo J.A., Wang A., Loftus P., Evans K.H., Chu I., Shieh L. (2014). Smarter hospital communication: Secure smartphone text messaging improves provider satisfaction and perception of efficacy, workflow. J. Hosp. Med..

[7] Tatum W.O. (2017). Ambulatory EEG Monitoring.

[8] MacGregor J.C., Oliver C.L., MacQuarrie B.J., Wathen C.N. (2021). Intimate partner violence and work: A scoping review of published research. Trauma Violence Abus..

[9] Postmus J.L., Hoge G.L., Breckenridge J., Sharp-Jeffs N., Chung D. (2020). Economic abuse as an invisible form of domestic violence: A multicountry review. Trauma Violence Abus..

[10] Gonzalez S. Unable to Leave: Economic Sabotage and Exploitation in Abusive Relationships. Future Without Violence, 2018. https://workplacesrespond.org/resource-library/.

[11] Postmus J.L., Plummer S.B., Stylianou A.M. (2016). Measuring economic abuse in the lives of survivors: Revising the Scale of Economic Abuse. Violence Against Women.

[12] Adams A.E., Sullivan C.M., Bybee D., Greeson M.R. (2011). Development of the scale of economic abuse. Violence Against Women.

[13] National Coalition Against Domestic Violence (2017). Quick Guide: Economic and Financial Abuse. https://ncadv.org/blog/posts/quick-guide-economic-and-financial-abuse#:~:text=Six%20Quick%20Economic%20Abuse%20Statistics&text=Between%2094%2D99%25%20of%20domestic,reasons%20stemming%20from%20the%20abuse.

[14] Postmus J.L., Hetling A., Hoge L. (2015). Evaluating a financial education curriculum as an intervention to improve financial behaviors and financial well-being of survivors of domestic violence: Results from a longitudinal randomized controlled study. J. Consum. Aff..

[15] Stylianou A.M. (2018). Economic abuse within intimate partner violence: A review of the literature. Violence Vict..

[16] Christy-McMullin K. (2003). Asset-building policies and safety for women: Stretching social work’s conceptual framework. Soc. Policy J..

[17] Johnson L., Chen Y., Stylianou A., Arnold A. (2022). Examining the impact of economic abuse on survivors of intimate partner violence: A scoping review. BMC Public Health.

[18] Sanders C.K., Schnabel M. (2006). Organizing for economic empowerment of battered women: Women’s savings accounts. J. Community Pract..

[19] Sauber E.W., O’Brien K.M. (2020). Multiple losses: The psychological and economic well-being of survivors of intimate partner violence. J. Interpers. Violence.

[20] Postmus J.L., Plummer S.B., McMahon S., Murshid N.S., Kim M.S. (2012). Understanding economic abuse in the lives of survivors. J. Interpers. Violence.

[21] Adams A.E., Sullivan C.M., Bybee D., Greeson M.R. (2008). Development of the Scale of Economic Abuse. Violence Against Women.

[22] Voth Schrag R.J., Edmond T. (2017). School sabotage as a form of intimate partner violence: Provider perspectives. Affilia.

[23] Showalter K., Bosetti R. (2021). The IPV-WDA: Developing an abusive partner-initiated workplace disruptions assessment using item response theory. J. Fam. Violence.

[24] Logan T.K., Cole J. (2011). Exploring the intersection of partner stalking and sexual abuse. Violence Against Women.

[25] Tenkorang E.Y., Owusu A.Y. (2019). Does economic abuse affect the health outcomes of women in Ghana?. Health Educ. Behav..

[26] Gürkan Ö.C., Ekşi Z., Deniz D., Çırçır H. (2020). The influence of intimate partner violence on pregnancy symptoms. J. Interpers. Violence.

[27] Showalter K., Choi M.S., Marcal K., Machinga R. (2023). Intimate Partner Violence: Understanding Employment Stability through Latent Class Analysis. Community Work. Fam..

[28] Tolman R.M., Wang H.C. (2005). Domestic violence and women’s employment: Fixed effects models of three waves of women’s employment study data. Am. J. Community Psychol..

[29] Yau J.H.Y., Wong J.Y.H., Fong D.Y.T. (2021). Economic abuse as a form of intimate partner violence: A literature review of the instruments and mental well-being outcomes. Violence Vict..

[30] Straus M.A., Hamby S.L., Boney-McCoy S.U.E., Sugarman D.B. (1996). The revised Conflict Tactics Scales (CTS2) development and preliminary psychometric data. J. Fam. Issues.

[31] Costa D., Barros H. (2016). Instruments to assess intimate partner violence: A scoping review of the literature. Violence Vict..

[32] Shepard M.F., Campbell J.A. (1992). The Abusive Behavior Inventory: A measure of psychological and physical abuse. J. Interpers. Violence.

[33] Tolman R.M. (1998). The development of a measure of psychological maltreatment of women by their male partners. Violence Vict..

[34] Hartley C.C., Renner L.M. (2018). Economic self-sufficiency among women who experienced intimate partner violence and received civil legal services. J. Fam. Violence.

[35] Weaver T.L., Sanders C.K., Campbell C.L., Schnabel M. (2009). Development and preliminary psychometric evaluation of the Domestic Violence—Related Financial Issues Scale (DV-FI). J. Interpers. Violence.

[36] Hegarty K., Bush R., Sheehan M. (2005). The Composite Abuse Scale: Further development and assessment of reliability and validity of a multidimensional partner abuse measure in clinical settings. Violence Vict..

[37] Campbell D.W., Campbell J., King C., Parker B., Ryan J. (1994). The reliability and factor structure of the Index of Spouse Abuse with African-American women. Violence Vict..

[38] Lehmann P., Simmons C.A., Pillai V.K. (2012). The validation of the Checklist of Controlling Behaviors (CCB) assessing coercive control in abusive relationships. Violence Against Women.

[39] Riger S., Ahrens C., Blickenstaff A. (2000). Measuring interference with employment and education reported by women with abusive partners: Preliminary data. Violence Vict..

[40] Brush L.D. (2002). Work-related abuse: A replication, new items, and persistent questions. Violence Vict..

[41] Bellini R., Lee K., Brown M.A., Shaffer J., Bhalerao R., Ristenpart T. The {digital-safety} risks of financial technologies for survivors of intimate partner violence. Proceedings of the 32nd USENIX Security Symposium (USENIX Security 23).

[42] U.S. Bureau of Labor Statistics (2024). U.S. Department of Labor, Occupational Outlook Handbook, Nurse Anesthetists, Nurse Midwives, and Nurse Practitioners. https://www.bls.gov/ooh/healthcare/nurse-anesthetists-nurse-midwives-and-nurse-practitioners.htm.

[43] Catalano S.M. (2013). Intimate Partner Violence–Attributes of Victimization, 1993–2011.

[44] Eisikovits Z., Winstok Z., Fishman G. (2004). The first Israeli national survey on domestic violence. Violence Against Women.

[45] Sharma K.K., Vatsa M. (2011). Domestic violence against nurses by their marital partners: A facility-based study at a tertiary care hospital. Indian J. Community Med..

[46] Da Rosa P., Brown R., Pravecek B., Carotta C., Garcia A.S., Carson P., Callies D., Vukovich M. (2021). Factors associated with nurses emotional distress during the COVID-19 pandemic. Appl. Nurs. Res..

[47] Zheng R., Zhou Y., Qiu M., Yan Y., Yue J., Yu L., Lei X., Tu D., Hu Y. (2021). Prevalence and associated factors of depression, anxiety, and stress among Hubei pediatric nurses during COVID-19 pandemic. Compr. Psychiatry.

[48] Arnetz J.E., Goetz C.M., Arnetz B.B., Arble E. (2020). Nurse reports of stressful Situations during the COVID-19 pandemic: Qualitative analysis of survey responses. Int. J. Environ. Res. Public Health.

[49] Ellsberg M., Emmelin M. (2014). Intimate partner violence and mental health. Glob. Health Action.

[50] Galanis P., Vraka I., Fragkou D., Bilali A., Kaitelidou D. (2021). Impact of personal protective equipment use on health care workers’ physical health during the COVID-19 pandemic: A systematic review and meta-analysis. Am. J. Infect. Control.

[51] Barrett D., Heale R. (2021). COVID-19: Reflections on its impact on nursing. Evid.-Based Nurs..

[52] Freed D., Palmer J., Minchala D., Levy K., Ristenpart T., Dell N. “A stalker’s paradise” How intimate partner abusers exploit technology. Proceedings of the 2018 CHI Conference on Human Factors in Computing Systems.

[53] Al-Modallal H. (2016). Effect of intimate partner violence on the health of women of Palestinian origin. Int. Nurs. Rev..

[54] Costello A.B., Osborne J. (2019). Best practices in exploratory factor analysis: Four recommendations for getting the most from your analysis. Pract. Assess. Res. Eval..

[55] Worthington R.L., Whittaker T.A. (2006). Scale development research: A content analysis and recommendations for best practices. Couns. Psychol..

[56] Boateng G.O., Neilands T.B., Frongillo E.A., Melgar-Quiñonez H.R., Young S.L. (2018). Best practices for developing and validating scales for health, social, and behavioral research: A primer. Front. Public Health.

[57] Showalter K., McCloskey R.J. (2021). A qualitative study of intimate partner violence and employment instability. J. Interpers. Violence.

[58] De Vellis R.F., Dancer L.S. (2016). Scale development: Theory and applications. J. Educ. Meas..

[59] Trochim W.M. (2006). Qualitative Validity. http://www.socialresearchmethods.net/kb/qualmeth.php.

[60] Graham J.W. (2009). Missing data analysis: Making it work in the real world. Annu. Rev. Psychol..

[61] Goretzko D., Pham T.T.H., Bühner M. (2019). Exploratory factor analysis: Current use, methodological developments and recommendations for good practice. Curr. Psychol..

[62] Arifin W.N. (2015). The graphical assessment of multivariate normality using SPSS. Educ. Med. J..

[63] Bentler P.M. (2006). EQS 6 Structural Equations Program Manual.

[64] Brown T.A. (2015). Confirmatory Factor Analysis for Applied Research.

[65] Courtney M.G.R. (2013). Determining the number of factors to retain in EFA: Using the SPSS r-menu v2. 0 to make more judicious estimations. Pract. Assess. Res. Eval..

[66] Fabrigar L.R., Wegener D.T. (2011). Exploratory Factor Analysis.

[67] Nguyenm H.V., Waller N.G. (2022). Local Minima and Factor Rotations in Exploratory Factor Analysis. Psychol. Methods.

[68] Hair J.F., Black W.C., Babin B.J., Anderson R.E. (2010). Multivariate Data Analysis.

[69] RStudio Team (2020). RStudio: Integrated Development for R. RStudio.

[70] Swanberg J., Macke C., Logan T.K. (2007). Working women making it work: Intimate partner violence, employment, and workplace support. J. Interpers. Violence.

[71] Showalter K. (2016). Women’s employment and domestic violence: A review of the literature. Aggress. Violent Behav..

[72] Whitlow M.L., Drake E., Tullmann D., Hoke G., Barth D. (2014). Bringing technology to the bedside: Using smartphones to improve interprofessional communication. Comput. Inform. Nurs..

[73] Duerksen K.N., Woodin E.M. (2019). Technological intimate partner violence: Exploring technology-related perpetration factors and overlap with in-person intimate partner violence. Comput. Hum. Behav..

[74] Kim C., Ferraresso R. (2023). Examining technology-facilitated intimate partner violence: A systematic review of journal articles. Trauma Violence Abus..

[75] Swanberg J.E., Ojha M.U., Macke C. (2012). State employment protection statutes for victims of domestic violence: Public policy’s response to domestic violence as an employment matter. J. Interpers. Violence.

[76] Pachner T.M., Showalter K., Maffett P. (2021). The Effects of Workplace Support on Workplace Disruptions: Differences Between White and Black Survivors of Intimate Partner Violence. Violence Against Women.

